# Fabrication of Copper Matrix Composites Reinforced with Carbon Nanotubes Using an Innovational Self-Reduction Molecular-Level-Mixing Method

**DOI:** 10.3390/ma15186488

**Published:** 2022-09-19

**Authors:** Bin Ya, Yang Xu, Linggang Meng, Bingwen Zhou, Junfei Zhao, Xi Chen, Xingguo Zhang

**Affiliations:** 1 Key Laboratory of Solidification Control and Digital Preparation Technology (Liaoning Province), Dalian University of Technology, Dalian 116024, China; 2 Ningbo Institute of Dalian University of Technology, Ningbo 315000, China

**Keywords:** carbon nanotube, copper matrix composite, molecular-level mixing, self-reduction, mechanical and electrical conductivity

## Abstract

An innovational self-reduction molecular-level-mixing method was proposed as a simplified manufacturing technique for the production of carbon nanotube copper matrix composites (CNT/Cu). Copper matrix composites reinforced with varying amounts of (0.1, 0.3, 0.5 and 0.7 wt%) carbon nanotubes were fabricated by using this method combined with hot-pressing sintering technology. The surface structure and elemental distribution during the preparation of CNT/Cu mixing powder were investigated. The microstructure and comprehensive properties of the CNT/Cu composites were examined by metallography, mechanical and electrical conductivity tests. The results revealed that the CNT/Cu could be produced by a high temperature reaction at 900 degrees under vacuum, during which the carbon atoms in the carbon nanotubes reduced the divalent copper on the surface to zero-valent copper monomers. The decrease in the ratio of D and G peaks on the Raman spectra indicated that the defective spots on the carbon nanotubes were wrapped and covered by the copper atoms after a self-reduction reaction. The prepared CNT/Cu powders were uniformly embedded in the grain boundaries of the copper matrix materials and effectively hindered the tensile fracture. The overall characteristics of the CNT/Cu composites steadily increased with increasing CNT until the maximum at 0.7 wt%. The performance was achieved with a hardness of 86.1 HV, an electrical conductivity of 81.8% IACS, and tensile strength of 227.5 MPa.

## 1. Introduction

Copper plays an essential function in life because of its good electrical and thermal properties and is extensively applied in electronics, electrical, construction, military and other fields [1]. Accordingly, an increasing amount of research is being devoted to the exploitation of copper-based composite [2,3] materials. A nanomaterial called a carbon nanotube (CNT) [4,5] has remarkable mechanical, electrical and thermal properties [6,7] due to its high strength and specific modulus. Therefore, it is an excellent reinforcing phase for the fabrication of new copper-based composites, and it has great potential in its application prospects [8]. Combining the features of these two materials, carbon nanotube copper matrix composites (CNT/Cu) [9] present a series of advantages, such as good mechanical hardness [10,11], electrical [12,13,14] and thermal conductivity [15,16], friction and wear resistance [17,18], and compression and tensile resistance [19,20,21] properties. It has been reported that the resulting microstructural arrays have an important role upon the material’s distinctive properties [22]. Additionally, the distributed particles also have positive effects on the resulting mechanical behavior of the composites [23].

However, the poor interfacial wetting ability of CNT and the clustering effect in the metal matrix limit the application of CNT in composites. The presence of defects such as inclusions and pores in clusters can degrade the performance of the composites. Recently, compared to the conventional preparation process, it has been well demonstrated that the molecular level mixing (MLM) method [24,25,26] successfully improved the interfacial bonding [27,28] and dispersion [29] of CNT with substrates at the molecular level by using surface modification [30]. Unfortunately, the requirement of using hydrogen as a reducing agent [31,32] in a variety of studies is accompanied by serious safety hazards and a complicated preparation process. A more convenient and efficient restoration method has not yet been studied.

In this paper, an innovative molecular-level-mixing self-reduction method was proposed and validated. Utilizing the reducibility of carbon atoms, the copper oxide on the surface of CNT was reduced to copper under high temperature and vacuum conditions. The feasibility and regularity of the self-reduction process was investigated and discussed through verification of characterizing CNT for different stages of the process by X-ray diffraction (XRD), infrared spectroscopy, optical microscopy, scanning electron microscope (SEM) and transmission electron microscope (TEM). The molecular-level-dispersed CNT/Cu composites with different CNT contents were successfully prepared by ball-milling [33,34] and hot-pressing processes [35,36]. The conductivity, mechanical properties and tensile behavior [37] of the fabricated composites were tested and analyzed.

## 2. Experimental

### 2.1. Reagents and Raw Materials

Carboxylated multi-walled carbon nanotubes (diameter: 10–20 nm, length: 10–30 μm, purity > 95%, density 2.1 g/cm^3^, from Nanjing Xianfeng Nanomaterials Technology, Nanjing, China), copper powder (particle size 300 nm, purity 99.9%, density 8.92 g/cm^3^, provided by Zhongzhi Xindun Alloy, Xingtai, China), Cupric acetate monohydrate (C_4_H_6_CuO_4_·H_2_O, purity 99.0%, from Tianjin Damao Chemical Reagent Factory, Tianjin, China), and alcohol (CH_3_CH_2_OH, ≥99.7%, density 0.789–0.791 g/mL, from TianDa Chemical Reagent Company, Tianjin, China) were obtained.

### 2.2. Preparation Procedure

**Figure 1:**
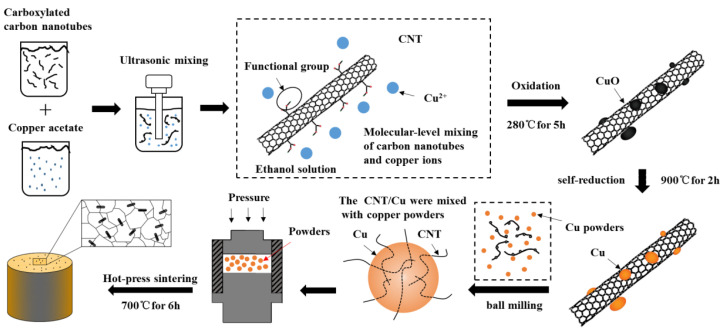
Principle of molecular-level-mixing method.

The CNT/Cu powder was subsequently obtained by the specific experimental schematic in Figure 1. Firstly, carboxylated multi-walled carbon nanotubes were mixed with saturated copper acetate alcohol solution and sonicated for 2 h by an Ultrasonic Cleaner (model: YL-060ST). Then, the suspension was filtered and dried at 80 °C (±2 °C) for 24 h. After grinding, the powder was oxidized at 280 °C (±5 °C) for 5 h to obtain carbon nanotube/copper oxide (CNT/CuO) powder. Finally, the CNT/CuO powder was subjected to self-reduction reactions under vacuum conditions at 80, 280, 800, and 900 degrees with a holding time of 2 h. The equipment used was a vacuum melting furnace (model: HZCZ-240).

The CNT/Cu composites were produced by using the mechanical ball-milling method and vacuum hot-pressing method. The prepared CNT/Cu powder and copper powder were put into a planetary ball mill (model: QM-3SP4, from Nanjing University Instrument Factory, Nanjing, China) with a ball-to-material ratio of 5:1. The rotating speed was 200 r/min. After 4 h of mechanical ball milling, a uniformly mixed composite powder was obtained. After that, the powder was heated to a temperature of 700 °C (±10 °C) at a heating rate of 10 °C/min and sintered at a pressure of 30 MPa. The holding period was 6 h. Finally, four groups of CNT/Cu composites were completed, containing CNT at 0.1, 0.3, 0.5, and 0.7 wt%.

### 2.3. Characterization

A Fourier transform near-infrared spectrometer (model: MPA0304040) and Laser confocal micro-Raman spectrometer (inVia Qontor 03040405) were used to examine the molecular structure and the structural integrity of the surface of the composite powders at different stages of treatment. CNTs/CuO and carbon nanotube powder samples were analyzed for the physical phase of the samples using an X-ray diffractometer (model: XRD-600003030502). The morphology of the CNTs/Cu composite samples were evaluated by metallographic microscopy (Leica DMi8) and the hardness, compression and tensile properties of the composites were tested by using a universal testing machine (CSS-44200). Five data points for hardness and conductivity were taken and averaged. The length of the tensile specimen was 40 mm, the width was 10 mm, and the thickness was 3 mm. The tensile test was performed at a strain rate of 3.7 × 10^−4^ s^−^^1^. Molecular binding of copper to carbon nanotubes at the microscopic level was observed by using field emission transmission electron microscope (JEM-2100F, from JEOL, Tokyo, Japan) and field emission scanning electron microscopy (SU5000, from HITAXHI, Tokyo, Japan).

## 3. Results and Analysis

### 3.1. Characterization of CNT/Cu Composite Powder

**Figure 2:**
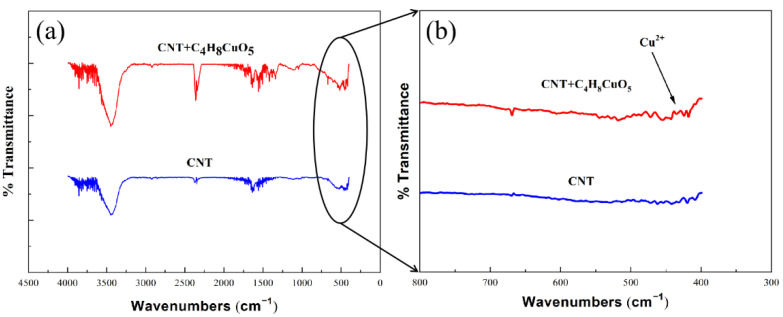
(**a**) Infrared spectra of powder and raw CNT powder after sonication; (**b**) Magnified band pictures of the spectra at 300–800 cm^−1^.

Figure 2 shows the comparison of the IR spectra of the powders in two states. One is the carbon nanotube powder mixed with saturated copper acetate alcohol solution after ultrasonic treatment (CNT + C_4_H_8_CuO_5_), and the other is the original carbon nanotube powder (CNT). From the figure, it can be observed that CNT + C_4_H_8_CuO_5_ exhibited an obvious peak segment of Cu^2+^ ions after sonication. This situation proves that the combination of Cu^2+^ ions and functional groups on the surface of CNT could be achieved by using ultrasonication. 

**Figure 3:**
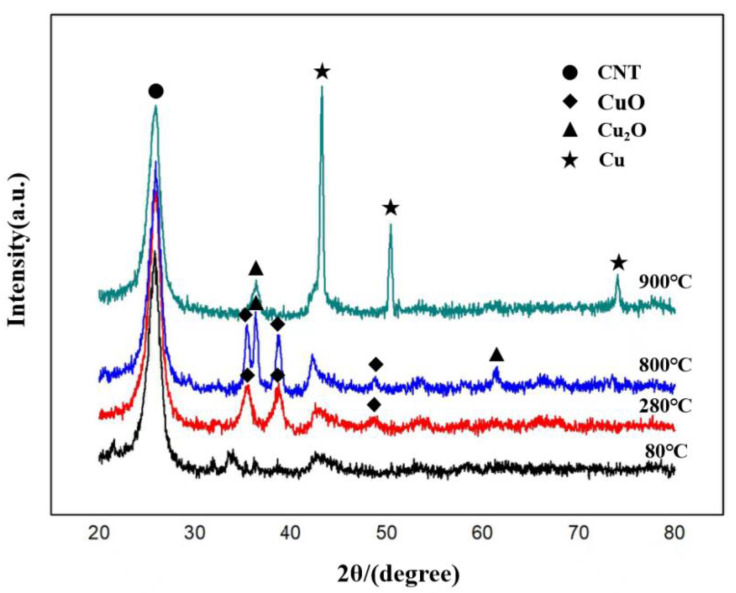
XRD patterns of the composites at different reduction temperatures (80 °C, 280 °C, 800 °C, 900 °C). The PDF file numbers are as follows. Copper (Cu): PDF#89-2838; Copper Oxide (Cu_2_O): PDF#77-0199; Cuprite, syn (CuO): PDF#78-0428;.

The XRD patterns of the samples of CNT/Cu powder at different stages of the self-reducing molecular-level-mixing process are shown in Figure 3. The corresponding peak of CNT (002) at 2θ = 25.9° appeared in all four groups of samples. The structure of CNT remained in a stable state and was not disrupted under the low to high temperature process conditions. After oxidization at 280 °C, the diffraction peak of CuO appeared on the curve in addition to the diffraction peak of CNT; the diffraction peak of CuO still existed in the plot when the reduction temperature was 800 °C, but the diffraction peak of Cu_2_O also existed, which means that the reduction process had already begun, and divalent copper had been partially reduced to univalent copper; the XRD plot displayed the diffraction peak of a copper monomer when the self-reduction reaction temperature was 900 °C. With the increase in temperature, the metal structure attached to the surface of CNT reacted from divalent Cu ions to Cu. It was apparent that the temperature strongly influenced the formation of the powders. The self-reduction method was feasible fpr achieving the molecular-level mixing of carbon nanotubes and copper, but it needed to reach a sufficiently high temperature because the reaction did not proceed sufficiently at temperatures below 900 °C. Therefore, the obvious self-reduction reaction appeared only when the temperature reached at least 900 °C. 

**Figure 4:**
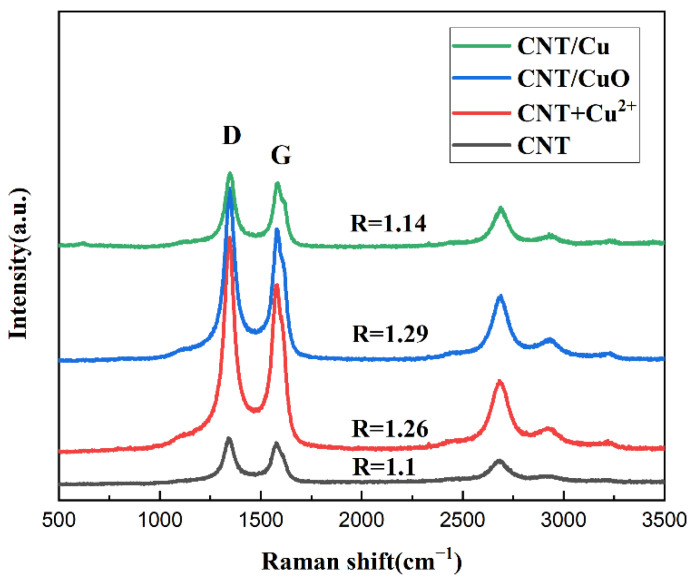
Raman spectra of sample powders processed after raw powder, ultrasonic mixing, oxidation, and self-reduction.

The four groups of sample powders with different treatments were analyzed by Raman spectroscopy, and the results are shown in Figure 4. R is the ratio of I_D_/I_G_, which means the intensity ratio between the D-peak and G-peak in the Raman spectra. This ratio can be used to describe the intensity relationship between these two peaks. A larger ratio indicates that the more defects there are in the CNT, the more unstable the structure. It can be seen from the figure that the ratio of the peaks of carboxylated CNT was the lowest, and the ratio increased gradually with the sonication and oxidation treatment. This is attributed to the damage of the CNT surface by the shock in the sonication. Alternatively, Cu^2+^ binds to functional groups at the molecular level resulting in defect problems due to the uneven alignment of carbon atoms. However, the R value of the powders after self-reduction reaction decreased instead. It can be inferred that the defective spots after the original surface treatment were wrapped and uniformly covered by the copper. This is the primary reason for the decrease in the intensity ratio of the D and G peaks.

**Figure 5:**
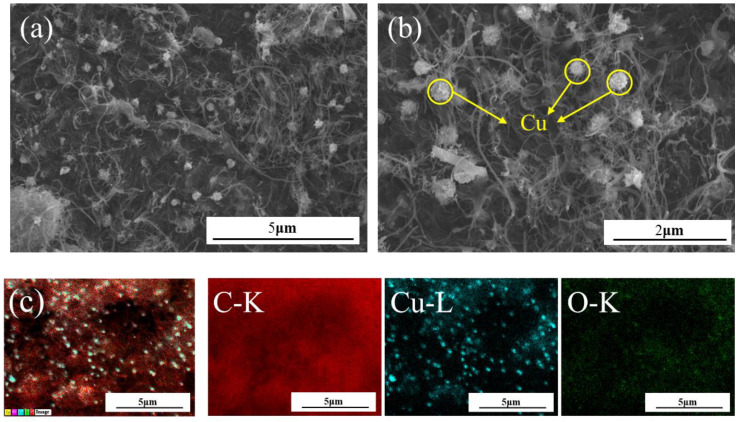
SEM morphology image of CNT/Cu composite powder (**a**) 10,000× (**b**) 20,000× (**c**) The EDS layered energy spectrum image, where the main components are Cu (blue), O (green), and C (red). It can be observed that a large area was covered by carbon, accounting for 90%. The uniformly attached spherical clusters are Cu elements, accounting for about 8%. The surface of the CNT was slightly oxidized, accounting for about 2%.

**Figure 6:**
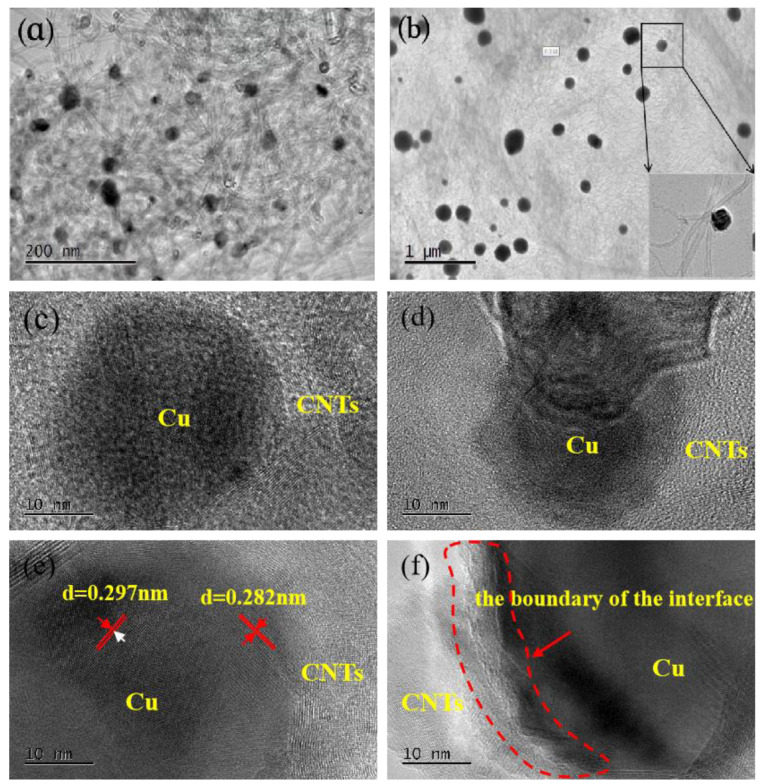
TEM images of CNT/Cu composite powder at (**a**,**b**) 900 °C (**c**) Copper atoms under multiple carbon tubes. Neatly arranged and uniformly oriented carbon tube stripes against a white background. (**d**) Black round copper on a bent carbon tube uniformly wrapped around the surface. (**e**,**f**) The boundary junction between copper and carbon tube was tightly fitted, with large lattice stripes of copper clearly visible.

Figure 5 and Figure 6 illustrate the microscopic morphology of the CNT/Cu composite powder obtained by reduction at a temperature of 900 °C. Spherical substances with diameters of 200–400 nm were attached along the surface of CNT, which were analyzed by energy spectroscopy (Figure 5c) as copper monomers. In addition, it was visible that the deposited copper particles in Figure 6a,b gathered together to form spherical metal particles. These particles were attached to the surface of CNT, forming a point-like distribution structure. As previously judged, when the reduction reaction of carbon atoms with copper oxide occured at high temperature, the surface functional groups gradually wetted the surface. The produced copper partially wrapped the dotted sites of carbon atoms after the reaction (Figure 6d). The interface between the copper and carbon was well bonded and tightly connected (Figure 6f). Moreover, the lattice stripes with inconsistent orientation directions of metallic Cu can be observed through Figure 6e. By analyzing and comparing the SEM and TEM images, it was confirmed that the molecular-level self-reduction reaction at 900 °C could be used to obtain CNT/Cu powders with good interfacial bonding. 

### 3.2. Morphology and Mechanical Properties of CNT/Cu Composites

**Figure 7:**
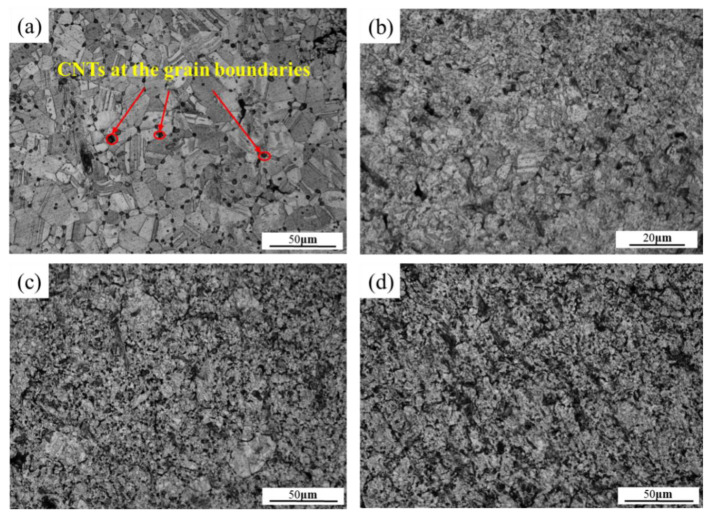
Metallographic pictures of surface-modified carbon nanotube reinforced copper matrix composites with different CNT contents of (**a**) 0.1 wt%; (**b**) 0.3 wt%; (**c**) 0.5 wt%; (**d**) 0.7 wt%.

The microstructures of the CNT-reinforced copper matrix composites with different contents of CNT by using a combination of the molecular-level-mixing self-reduction method and hot-pressing process are shown in Figure 7. The well-defined grain boundaries and complete coarse grains are obviously visible in Figure 7a. The black dot-like distribution of the CNT particles is regularly and uniformly dispersed and embedded in the grain boundaries. On the contrary, the grains were refined with the increase in CNT content (Figure 7b,c). However, black-phase carbon aggregation appeared with increasing the content of CNT (Figure 7d). Furthermore, pores, inclusions, and cracking defects tended to appear at the locations where carbon nanotubes were aggregated. Therefore, the results indicate that a limited amount of CNT could effectively refine the grains, while an excessive increase in the content of CNT leads to aggregation. This problem can be alleviated by adjusting the ball-milling process.

According to the density formula of composite materials as shown in the equation,

$$\rho_{c}=\frac{\rho_{m}\rho_{f}}{w_{m}\rho_{f}+\left(1-w_{m}\right)\rho_{m}}$$

where $\rho_{c}$: density of the composite material; $\rho_{m}$: density of the substrate; $\rho_{f}$: density of the enhanced system; and $w_{m}$: Mass fraction of matrix in the composite material, theoretically, it is known that the higher the CNT content, the lower the density of the material. When the CNT content was 0.1 wt%, 0.3 wt% and 0.5 wt%, the CNT was distributed at the grain boundaries, refining the metal grains and making the structure compact. As a result, the density gradually increased. However, when the CNT content reached 0.7 wt%, the high content of CNT was more likely to agglomerate, leading to some tiny pores in the middle of the material, which made a slight decrease in density. 

**Figure 8:**
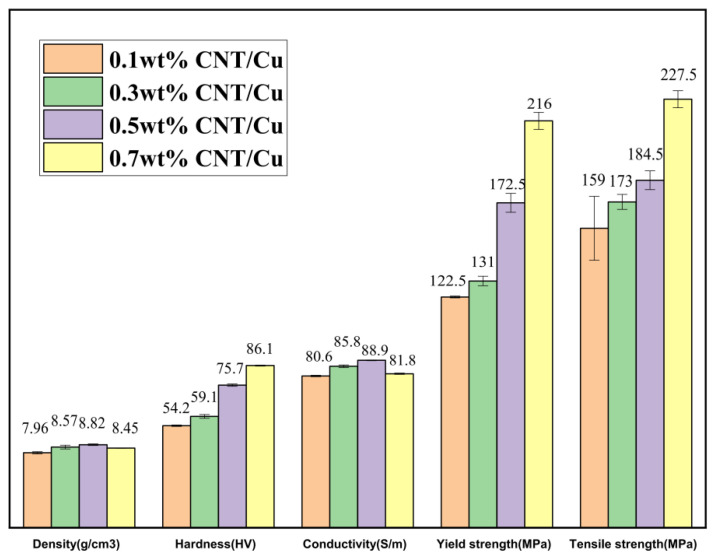
Histogram of density, hardness, conductivity, yield strength, and tensile strength of samples with different CNT content of 0.1 wt%, 0.3 wt%, 0.5 wt%, and 0.7 wt%.

The results shown in Figure 8 visually represent that the hardness increased rapidly with the increase in CNT content, reaching a maximum hardness of 86.1 HV at 0.7 wt%, which was 58.9% higher than that at 0.1 wt%. The carbon nanotubes as reinforcing materials are equivalent to “fibers” firmly adhered to the inside of the matrix, hindering the dislocation movement and enhancing the performance of the composite. Although the conductivity continued to increase when the CNT content increased from 0.1 wt% to 0.3 wt%, the conductivity decreased slightly at 0.7 wt% due to the increased porosity of the composites. Defects hinder electron migration, which largely affects the conductivity of the copper matrix composites. It can be seen that there was a regular increase in yield strength and tensile strength with increasing the CNT content. The performance surges and elongation were the best at 0.7 wt%. 

**Figure 9:**
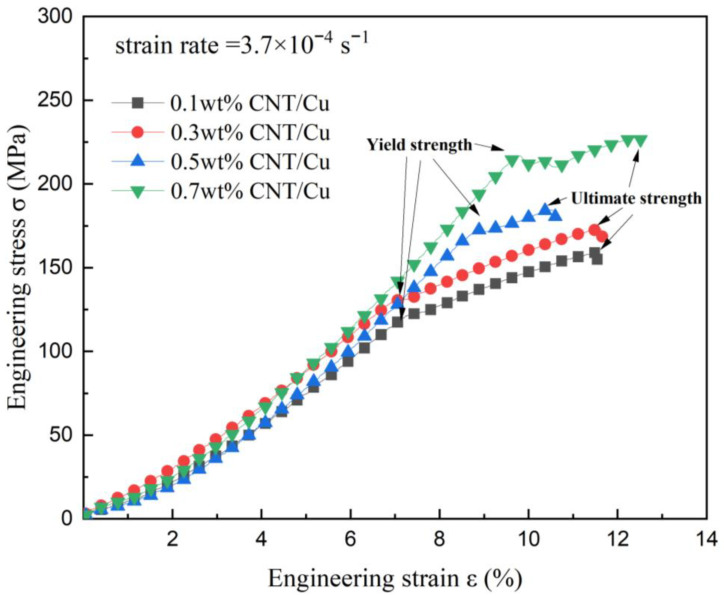
Stress–strain curve of samples with different CNT content of 0.1 wt%, 0.3 wt%, 0.5 wt%, and 0.7 wt%.

However, the low elongation at 0.5 wt% of the carbon nanotube content in the stress–strain curve (Figure 9) highlights the problem that the poor dispersion of CNT in the metal matrix lead to increased agglomeration phenomena and defects in a small area. The sample had difficulty in withstanding higher tensile forces and fractured prematurely, so that the plasticity of the material decreased significantly. By optimizing the hot-pressing process, the dispersion of CNT and the properties of CNT/Cu composites could be further improved.

**Figure 10:**
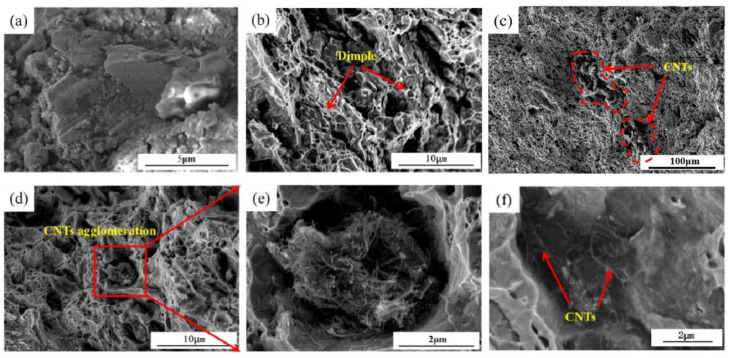
Fracture morphology of CNT/Cu composites with different CNT content of (**a**) 0.1 wt%; (**b**) 0.3 wt%; (**c**) 0.5 wt%; (**d**–**f**) 0.7 wt%.

Figure 10 shows the morphological characteristics of the fracture of the tensile sample. At a CNT content of only 0.1 wt%, the fracture level was a relatively flat plane interlocking and no apparent presence of CNT can be observed (Figure 10a). When the content of CNT increased to 0.3 wt%, the fracture morphology appeared to be an obvious microporous aggregation type ductile fracture with unevenness (Figure 10b). The microscopic morphology of the fracture was honeycomb-like, and the fracture surface consisted of a significant amount of tiny tough nests, which were about 1μm black nest pits (Figure 10c). A large number of CNT was found to be aggregated at the fracture site as the content increased (Figure 10d), with some clustered together (Figure 10e). The agglomerated carbon nanotubes were defective weak parts in the material, which were easy to fracture. The fracture surface had CNT agglomeration and entanglement phenomena. Plenty of CNT extended inside the matrix to bear the load through the interior of the matrix (Figure 10e), which effectively increased the tensile strength.

## 4. Conclusions

(1) By performing the self-reduction reaction of CNT/CuO at different temperatures, it was shown that the preparation of CNT/Cu could be achieved under vacuum at 900 °C. The copper bonding was tight on the surface of CNT without damage to the overall structure.
(2) With the increase in CNT content (0.1 wt%, 0.3 wt%, and 0.5 wt%), the copper matrix composites could progressively improve the density and electrical conductivity. However, the electrical conductivity and density of the composite with 0.7 wt% CNT content was reduced. The agglomeration defects were the most significant problem leading to cracking, which is detrimental to the performance. This problem can be alleviated by adjusting the ball-milling process.
(3) With the increase in CNT content (0.1 wt%, 0.3 wt%, 0.5 wt%, and 0.7 wt%), the composites could effectively resist mechanical forces. The highest elongation at break was achieved with a CNT content of 0.7 wt%. Both yield strength and ultimate strength were enhanced with a rise in CNT content from 0.1 wt% to 0.7 wt%.
(4) In summary, the CNT/Cu composite with 0.7 wt% CNT content had the best overall properties. The hardness reached 86.1 HV and the conductivity reached 87.8% IACS. The yield strength reached 216 MPa and ultimate strength reached 227.5 MPa.

## Data Availability

Required data is embedded within the article.

## References

[1] Yusoff M., Zuhailawati H. (2022). In Situ Tungsten Carbide Formation in Nanostructured Copper Matrix Composite Using Mechanical Alloying and Sintering. Materials.

[2] Chen F.Y., Ying J.M., Wang Y.F., Du S.Y., Liu Z.P., Huang Q. (2016). Effects of graphene content on the microstructure and properties of copper matrix composites. Carbon.

[3] Chen X., Bao R., Yi J., Fang D., Tao J., Liu Y. (2019). Enhancing Interfacial Bonding and Tensile Strength in CNT-Cu Composites by a Synergetic Method of Spraying Pyrolysis and Flake Powder Metallurgy. Materials.

[4] Iijima S. (1991). Helical microtubules of graphitic carbon. Nature.

[5] Sun X., Zeng X., Shu X., Cheng Z. (2001). Properties and applications of carbon nanotubes. China Powder Technol..

[6] Duong H.M., Tran T.Q., Kopp R., Myint S.M., Peng L. (2019). Direct Spinning of Horizontally Aligned Carbon Nanotube Fibers and Films From the Floating Catalyst Method. Nanotube Superfiber Materials.

[7] Duong H.M., Myint S.M., Tran T.Q., Le D.K. (2020). Post-spinning treatments to carbon nanotube fibers. Carbon Nanotube Fibers and Yarns.

[8] Sundaram R.M., Sekiguchi A., Sekiya M., Yamada T., Hata K. (2018). Copper/carbon nanotube composites: Research trends and outlook. R. Soc. Open Sci..

[9] Meng L., Wang X., Hu X., Shi H., Wu K. (2019). Role of structural parameters on strength-ductility combination of laminated carbon nanotubes/copper composites. Compos. Part A Appl. Sci. Manuf..

[10] Wei X., Tao J., Liu Y., Bao R., Li F., Fang D., Li C., Yi J. (2019). High strength and electrical conductivity of copper matrix composites reinforced by carbon nanotube-graphene oxide hybrids with hierarchical structure and nanoscale twins. Diam. Relat. Mater..

[11] Shuai J., Xiong L., Zhu L., Li W. (2016). Enhanced strength and excellent transport properties of a superaligned carbon nanotubes reinforced copper matrix laminar composite. Compos. Part A Appl. Sci. Manuf..

[12] Chen L., Hou Z., Liu Y., Luan C., Zhu L., Li W. (2020). High strength and high ductility copper matrix composite reinforced by graded distribution of carbon nanotubes. Compos. Part A Appl. Sci. Manuf..

[13] Milowska K.Z., Burda M., Wolanicka L., Bristowe P.D., Koziol K.K.K. (2018). Carbon nanotube functionalization as a route to enhancing the electrical and mechanical properties of Cu–CNT composites. Nanoscale.

[14] Fu S., Chen X., Liu P., Cui H., Zhou H., Ma F., Li W. (2022). Tribological Properties and Electrical Conductivity of Carbon Nanotube-Reinforced Copper Matrix Composites. J. Mater. Eng. Perform..

[15] Shaari N.S., Ismail M.H., Jumahat A., Zainudin M., Manap M.F.A., Shaari N. (2021). Thermal Conductivity of Copper Matrix Composites Reinforced with Multi-wall Carbon Nanotubes. J. Phys. Conf. Ser..

[16] Kim K.T., Eckert J., Liu G., Park J.M., Lim B.K., Hong S.H. (2011). Influence of embedded-carbon nanotubes on the thermal properties of copper matrix nanocomposites processed by molecular-level mixing. Scr. Mater..

[17] Murugesan R., Gopal M., Murali G. (2019). Effect of Cu, Ni addition on the CNTs dispersion, wear and thermal expansion behavior of Al-CNT composites by molecular mixing and mechanical alloying. Appl. Surf. Sci..

[18] Taşdemir F., Özyürek D., Yildirim M. (2019). Effect of Carbon Nanotube Content on the Wear Behaviours of Cu-CNT Composites Produced by Powder Metallurgy Method. Acta Phys. Pol. A.

[19] Nam D.H., Kim Y.K., Cha S.I., Hong S.H. (2012). Effect of CNTs on precipitation hardening behavior of CNT/Al–Cu composites. Carbon.

[20] Han B., Guo E., Xue X., Zhao Z., Luo L., Qu H., Niu T., Xu Y., Hou H. (2017). Fabrication and densification of high performance carbon nanotube/copper composite fibers. Carbon.

[21] Liu J., Fan G., Tan Z., Guo Q., Su Y., Li Z., Xiong D.-B. (2019). Mechanical properties and failure mechanisms at high temperature in carbon nanotube reinforced copper matrix nanolaminated composite. Compos. Part A Appl. Sci. Manuf..

[22] Rosa D.M., Spinelli J.E., Osório W.R., Garcia A. (2006). Effects of cell size and macrosegregation on the corrosion behavior of a dilute Pb–Sb alloy. J. Power Sources.

[23] Meyer Y.A., Bonatti R.S., Bortolozo A.D., Osório W.R. (2021). Electrochemical behavior and compressive strength of Al-Cu/xCu composites in NaCl solution. J. Solid State Electrochem..

[24] Cha S.I., Kim K.T., Arshad S.N., Mo C.B., Hong S.H. (2005). Extraordinary Strengthening Effect of Carbon Nanotubes in Metal-Matrix Nanocomposites Processed by Molecular-Level Mixing. Adv. Mater..

[25] Hwang J., Yoon T., Jin S.H., Lee J., Kim T.-S., Hong S.H., Jeon S. (2013). Enhanced Mechanical Properties of Graphene/Copper Nanocomposites Using a Molecular-Level Mixing Process. Adv. Mater..

[26] Cha S.I., Kim K.T., Lee K.H., Mo C.B., Hong S.H. (2005). Strengthening and toughening of carbon nanotube reinforced alumina nanocomposite fabricated by molecular level mixing process. Scr. Mater..

[27] Kim K.T., Eckert J., Menzel S.B., Gemming T., Hong S.H. (2008). Grain refinement assisted strengthening of carbon nanotube reinforced copper matrix nanocomposites. Appl. Phys. Lett..

[28] Daneshvar F., Zhang T., Aziz A., Sue H.-J., Welland M.E. (2020). Tuning the composition and morphology of carbon nanotube-copper interface. Carbon.

[29] Liu L., Bao R., Yi J., Li C., Tao J., Liu Y., Tan S., You X. (2017). Well-dispersion of CNTs and enhanced mechanical properties in CNTs/Cu-Ti composites fabricated by Molecular Level Mixing. J. Alloy. Compd..

[30] Hwang J.Y., Lim B.K., Tiley J., Banerjee R., Hong S.H. (2013). Interface analysis of ultra-high strength carbon nanotube/nickel composites processed by molecular level mixing. Carbon.

[31] Tu J.F., Rajule N., Molian P., Liu Y. (2016). Laser synthesis of a copper–single-walled carbon nanotube nanocomposite via molecular-level mixing and non-equilibrium solidification. J. Phys. D Appl. Phys..

[32] Lim B.K., Mo C.B., Nam D.H., Hong S.H. (2010). Mechanical and Electrical Properties of Carbon Nanotube/Cu Nanocomposites by Molecular-Level Mixing and Controlled Oxidation Process. J. Nanosci. Nanotechnol..

[33] Bor A., Ichinkhorloo B., Uyanga B., Lee J., Choi H. (2018). Cu/CNT nanocomposite fabrication with different raw material properties using a planetary ball milling process. Powder Technol..

[34] Singhal S.K., Lal M., Sharma I., Mathur R.B. (2012). Fabrication of copper matrix composites reinforced with carbon nanotubes using a combination of molecular-level-mixing and high energy ball milling. J. Compos. Mater..

[35] Babu R.V., Verma K.A., Charan M., Kanagaraj S. (2018). Tweaking the diameter and concentration of carbon nanotubes and sintering duration in Copper based composites for heat transfer applications. Adv. Powder Technol..

[36] Pham V.T., Bui H.T., Tran B.T., Nguyen V.T., Le D.Q., Than X.T., Doan D.P., Phan N.M. (2011). The effect of sintering temperature on the mechanical properties of a Cu/CNT nanocomposite prepared via a powder metallurgy method. Adv. Nat. Sci. Nanosci. Nanotechnol..

[37] Xue Z.W., Wang L.D., Zhao P.T., Xu S.C., Qi J.L., Fei W.D. (2012). Microstructures and tensile behavior of carbon nanotubes reinforced Cu matrix composites with molecular-level dispersion. Mater. Des..

